# Autophagy as a therapeutic target for cisplatin-resistant gastric cancer

**DOI:** 10.1016/j.gendis.2025.101992

**Published:** 2025-12-19

**Authors:** Luling Wei, Yingfei Zhou, Jiashuo Li, Hongzhao Qi, Shasha Wang

**Affiliations:** a Department of Oncology, The Affiliated Hospital of Qingdao University, Qingdao, Shandong 266000, China; b Institute for Translational Medicine, The Affiliated Hospital of Qingdao University, College of Medicine, Qingdao University, Qingdao, Shandong 266021, China

**Keywords:** Autophagy, Cisplatin, Drug resistance, Gastric cancer, Targeted therapy

## Abstract

Cisplatin is widely employed in the treatment of gastric cancer (GC). However, the resistance mechanisms exhibited by GC cells often result in suboptimal clinical outcomes associated with cisplatin therapy. Autophagy, a self-degradative cellular process, plays a complex dual role in regulating tumor cell death and survival. In recent years, significant attention has been directed toward the relationship between autophagy and cisplatin resistance in GC, fostering the development of various autophagy-related drugs and potential targets aimed at enhancing the sensitivity of GC cells to cisplatin. Nevertheless, a comprehensive analysis of the correlations among relevant studies is still lacking. This review synthesizes recent research examining the impact of autophagy on cisplatin resistance in GC cells, with particular emphasis on existing drugs and potential therapeutic drugs/targets. It briefly explores the fundamental processes of autophagy and clarifies the relationship between autophagy mechanisms and GC. Furthermore, it summarizes the available drugs and potential candidates that can either enhance or inhibit autophagy, thereby improving GC cell sensitivity to cisplatin, alongside their underlying mechanisms. Additionally, it consolidates pertinent research findings to present a more thorough understanding of the intricate relationships between autophagy and cisplatin resistance in GC cells. We hope this review will encourage researchers to investigate novel mechanisms of cisplatin resistance in GC cells, discover new targeted therapies, and propose innovative strategies to tackle this challenge.

## Introduction

Gastric cancer (GC) is the fifth most prevalent malignancy and the third leading cause of cancer-related deaths worldwide.^1^, ^2^, ^3^ In 2022, approximately 990,000 individuals were diagnosed with GC, resulting in around 740,000 deaths attributed to the disease.^4^ The highest incidence and mortality rates of GC were reported in East Asia, at 2.64% and 1.84%, respectively.^5^ Notably, China accounted for 44% of the global GC cases,^6^ underscoring the urgent need for effective prevention and treatment strategies for this cancer.

Cisplatin (DDP), a platinum-based chemotherapy agent,^7^ is the principal treatment for advanced GC.^8^ Its therapeutic effects on GC cells are multifaceted. The primary mechanism of action involves the formation of DNA crosslinks in cancer cells, which disrupts DNA replication and transcription processes, ultimately leading to DNA damage and the activation of cellular signaling pathways that induce cell cycle arrest and apoptosis.^9^^,^^10^ Through these mechanisms, DDP effectively inhibits the proliferation of GC cells and initiates programmed cell death. Furthermore, DDP can induce the production of reactive oxygen species (ROS) within cancer cells, resulting in oxidative stress that further compromises cellular integrity.^11^ This oxidative stress significantly contributes to the cytotoxicity of DDP in GC cells. However, the challenge of primary or acquired resistance presents a significant obstacle in DDP-based GC therapy, as various mechanisms contribute to this resistance. These mechanisms encompass enhanced DNA damage repair capacity, autophagy, epithelial–mesenchymal transition, decreased intracellular drug accumulation, inhibition of apoptosis, and elevated glutathione levels.^12^, ^13^, ^14^, ^15^ Consequently, addressing these specific resistance mechanisms with tailored treatments to restore the sensitivity of GC cells to DDP holds considerable and lasting clinical implications.

Autophagy is a complex catabolic process in which lysosomes degrade excessive or damaged organelles, proteins, and foreign extracellular bodies.^8^^,^^16^^,^^17^ This process plays a crucial role in regulating various aspects of tumorigenesis, including apoptosis, metastasis, and the cell cycle.^18^ Recent studies have highlighted the dual role of autophagy in mediating resistance to DDP in GC cells.^19^^,^^20^ On the one hand, the activation of autophagy in GC cells can lead to increased resistance to DDP. For instance, the overexpression of aquaporin 3 (AQP3) promotes autophagy, thereby enhancing the resistance of GC cells to DDP.^21^ Similarly, elevated levels of microfibrillar-associated protein 2 (MFAP2) induce autophagy in GC cells, further contributing to DDP resistance.^22^ Conversely, autophagy activation can also result in decreased resistance to DDP. For example, the inhibition of microRNA-21 (miR-21) can sensitize DDP-resistant GC cells by inducing autophagy.^23^ In conclusion, regardless of the underlying mechanisms, regulating autophagy represents a promising strategy to overcome DDP resistance in GC cells.

Conducting a systematic review of the field is essential as research advances to delineate future research directions and offer valuable insights for the development of related therapeutics. This review begins by summarizing the molecular mechanism of DDP resistance in GC cells (Table 1) and then provides a succinct overview of autophagy, highlighting its dual role in mediating DDP resistance in GC cells. Moreover, we summarize existing medications and potential drug candidates or targets that enhance the sensitivity of GC cells to DDP by either inducing or inhibiting autophagy, along with their underlying mechanisms. This approach aims to foster a more comprehensive understanding of the complex interactions between autophagy and DDP resistance in GC cells. Ultimately, this review seeks to inspire researchers to explore novel mechanisms of DDP resistance in GC cells, identify new targeted therapies, and propose innovative strategies to address this significant challenge.

**Table 1:** The molecular mechanism of cisplatin resistance in gastric cancer cells.

Mechanism category	Key molecules/factors	Function and resistance mechanisms	Reference
Enhancing DNA repair capacity	XRCC1, NER protein (XPA, XPB, XPC, CSB, XPD, XPF/ERCC1, ERCC2), RECQL4	(a) JWA/TXNL1 down-regulation → XRCC1 overexpression → enhanced DNA repair → enhanced drug resistance (b) miR-192-5p overexpression → targeted inhibition of ERCC3/4 → decreased NER ability → enhanced drug resistance (c) RECQL4 overexpression → YB1 phosphorylation increase → MDR1 overexpression → enhanced drug resistance	24, 25, 26, 27, 28, 29
Reducing cell apoptosis	CIP2A, miR-106a/PTEN, FOXO1, RPS3	(a) CIP2A overexpression → inhibition of apoptosis + MDR protein overexpression → enhanced drug resistance (b) miR-106a overexpression → PTEN down-regulation → PI3K/Akt activation (c) FOXO1 → PI3K/Akt activation → inhibition of apoptosis (d) RPS3→ activation of PI3K-Akt-cofilin-1 pathway → down-regulation of cofilin-1 mitochondrial translocation/PP1&PP2A → inhibition of apoptosis → enhanced drug resistance	30, 31, 32, 33, 34, 35
Promoting epithelial–mesenchymal transition (EMT)	HER2, HKDC1, MK, RIPK4	(a) HER2 up-regulation → EMT induction → enhanced drug resistance (b) HKDC1 overexpression → glycolysis increase + DNA damage increase → NF-κB activation → promotion of EMT → enhanced drug resistance (c) CAF-derived MK → lncRNA ST7-AS1 overexpression → PI3K/AKT activation → promotion of EMT (d) RIPK4 down-regulation in CAF → inhibition of PI3K/AKT → reduction of drug resistance	36, 37, 38, 39, 40
Regulating the tumor microenvironment	TAM (model 2), miR-21, miR-1911-5p, TMEM205	(a) M2 macrophage exosomes miR-21 → PTEN down-regulation → PI3K/AKT activation → inhibition of apoptosis (b) M2 exosomes miR-1911-5p → regulation of iron death by MYB/AKR1B10/ACC pathway → enhanced drug resistance (c) TMEM205 → TAM M2 polarization + Wnt/β-catenin activation → promotion of EMT/angiogenesis	3,41,42
Epigenetic regulation	MeCPc2/PDK-1, LINC-PINT/EZH2/ATG5	(a) MeCP2 → transcriptional activation of PDK-1 → activation of AKT pathway → enhanced drug resistance (b) LINC-PINT recruits EZH2 → inhibition of ATG5 promoter → inhibition of autophagy → reduction of drug resistance	43,44

## Autophagy and GC

### Overview of autophagy

In the 1950s, de Duve coined the term “autophagy” to describe a cellular self-degradation mechanism.^45^^,^^46^ Autophagy encompasses the process by which cytoplasmic components, damaged organelles, and misfolded proteins are sequestered within membranes to form autophagosomes, facilitating self-degradation and thereby maintaining cellular homeostasis.^47^ Important autophagy-related genes (ATGs) were identified in the 1990s. Autophagy is classified into three primary types: microautophagy, macroautophagy, and chaperone-mediated autophagy.^16^^,^^20^ While these pathways differ in morphology and mechanisms, they converge on lysosomal degradation.^48^ Notably, macroautophagy, commonly referred to simply as autophagy, is significantly implicated in human diseases.^49^^,^^50^ In the context of this review, the term “autophagy” specifically denotes “macroautophagy”, excluding the other two types. Autophagy plays a critical role in cellular defense. However, under adverse conditions, such as nutrient scarcity, excessive autophagic activity may contribute to cancer development due to self-degradation.^49^^,^^50^

**Figure 1:**
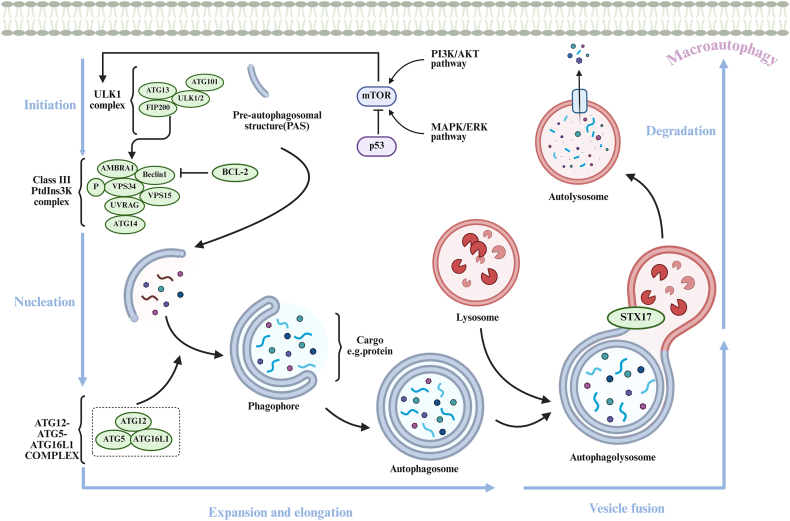
The process of autophagy. Autophagy consists of five stages: initiation, nucleation, expansion and elongation, vesicle fusion, and degradation. i) Initiation: The ULK1 complex induces nucleation at the PAS; ii) Nucleation: Phagophores are nucleated with the assistance of the PtdIns3K complex; iii) Expansion and elongation: Phagophores encircle proteins and other cargos to close the outer membrane and form autophagosomes; iv) Vesicle fusion: The STX17 protein facilitates the fusion of autophagosomes with lysosomes to create autophagolysosomes; v) Degradation: Acidic enzymes within autolysosomes degrade the cargo and transport the resulting metabolites to the cytoplasm.

The autophagic process comprises five sequential steps, as illustrated in Figure 1: initiation, vesicle nucleation, expansion and elongation, fusion of vesicles with lysosomes, and degradation of products, all of which are closely associated with ATGs.^51^ Autophagy is initiated by various stress signals, including nutrient deprivation, oxidative stress, and protein aggregation.^52^ The initiation phase involves the activation of the Unc-51-like kinase 1 (ULK1) complex, which consists of ULK1/2, the FAK family-interacting protein of 200 kDa (FIP200), ATG13, and ATG101.^51^^,^^52^ This complex is regulated by the nutrient-sensing mammalian target of rapamycin complex 1 (mTORC1), which suppresses autophagy under nutrient-rich conditions.^53^ Upon initiation, the ULK1 complex phosphorylates and activates the class III phosphatidylinositol 3-kinase (PtdIns3K, also known as PI3KC3), which comprises Beclin-1, vacuolar protein sorting 34 (VPS34), VPS15, and ATG14. This complex is responsible for generating phosphatidylinositol 3-phosphate (PI3P) on the phagophore, the initial membrane structure that encases the cargo for degradation. It is important to note that the regulation of the PtdIns3K complex primarily occurs through interactions with proteins associated with Beclin-1; additionally, the binding of Beclin-1 to the anti-apoptotic protein B cell lymphoma protein-2 (Bcl-2) influences the activity of PI3KC3.^49^ Subsequently, the phagophore expands and engulfs cytoplasmic contents to form a double-membraned vesicle known as the autophagosome. This stage involves the recruitment of various autophagy-related proteins, including the ATG12–ATG5–ATG16L1 complex, the class III PtdIns3K complex, light chain 3 (LC3)-II, and ATG9. LC3-II plays a crucial role in the elongation and sealing of the autophagosomal membrane. The maturation of the autophagosome involves its fusion with lysosomes, resulting in the formation of an autophagolysosome, a process that requires the SNARE protein syntaxin 17 (STX17) and the lysosomal-associated membrane protein 2 (LAMP2).^52^ Following fusion, lysosomal hydrolases degrade the engulfed cargo, releasing breakdown products such as amino acids, fatty acids, and sugars for cellular recycling.^54^^,^^55^

The regulatory mechanisms of autophagy are complex, involving not only autophagy-related proteins but also various signaling pathways, notably including the mTOR-dependent and mTOR-independent pathways.^53^ Among these, the phosphatidylinositol 3-kinase/mammalian target of rapamycin (PI3K/mTOR) and AMP-activated protein kinase (AMPK) pathways are particularly significant, serving as primary regulatory routes for autophagy.^56^ Under conditions of nutrient abundance, mTOR activation inhibits autophagy; conversely, during nutrient scarcity, mTOR activity decreases, thereby promoting autophagy. Furthermore, AMPK activation occurs in response to low energy levels, facilitating autophagy by inhibiting mTOR and activating ULK1.^56^ In conclusion, the current understanding of autophagy regulation emphasizes the roles of autophagy-related proteins and the key signaling pathways outlined above.

### The interplay between autophagy and GC

The intricate interplay between autophagy and GC involves a delicate balance of promoting and inhibitory factors, which are influenced by various cellular contexts and signaling pathways.^57^ Autophagy suppresses GC tumorigenesis by maintaining cellular integrity through selective clearance of damaged organelles and proteins, thereby preserving genomic stability and impeding cancer development.^58^ In addition, aberrant or excessive autophagic activity in GC cells can induce cytotoxicity by causing the improper degradation of essential intracellular components crucial for tumor cell survival, ultimately resulting in autophagic cell death and hindering tumor development.^57^^,^^59^ For instance, studies have demonstrated that 5-fluorouracil (5-FU) can up-regulate Beclin-1, enhancing autophagy, which in turn triggers autophagic cell death, ultimately inhibiting GC progression.^58^ Moreover, autophagy plays a pivotal role in modulating the immune response against cancer cells by promoting the presentation of tumor antigens to the immune system, thereby augmenting the recognition and elimination of malignant cells through immune effector mechanisms.

Conversely, autophagy can also facilitate GC progression through multiple mechanisms. One significant aspect is its ability to enhance the survival of tumor cells by increasing their stress tolerance and supplying essential nutrients.^57^^,^^60^ The tumor microenvironment provides the energy resources necessary for tumor cell metabolism and mitigates ROS production induced by treatment or drug metabolism. In this context, autophagy promotes tumor cell survival and may correlate with drug resistance.^60^ For instance, a study illustrated that activating autophagy through the phosphorylation of the protein kinase B (AKT) and subsequent inhibition of the PI3K/AKT/mTOR pathway, involving the P70 ribosomal protein S6 kinase 1 (P70S6K1), prevented apoptosis in GC MGC803 cells.^61^ Furthermore, autophagy contributes to drug resistance in GC cells. For example, the cytoprotective autophagy induced by oxaliplatin partially counteracted apoptotic cell death in GC MGC803 cells.^62^

In summary, the complex mechanisms by which autophagy promotes or inhibits GC are multifaceted and context-dependent, complicating the regulation of GC chemotherapy resistance by autophagy.

### Autophagy and DDP resistance in GC

Autophagy can manifest in multiple functional modes, mainly divided into cytoprotective autophagy that promotes cell survival and protection, and cytotoxic autophagy that promotes the death of tumor cells.^11^ In GC, autophagy demonstrates a high degree of environmental context-dependence, which may be specifically influenced by factors such as cell line type, the tumor microenvironment, disease stage, the abundance of autophagosomes, and external stimuli.^63^^,^^64^ Notably, it is closely associated with elements including functional p53 status, vitamin *D* treatment, drug sensitivity, and cancer progression.^65^ Although stratifying patients according to the nature of autophagy induction holds promise for improving therapeutic outcomes in specific patient subpopulations, there remain no definitive quantitative, biochemical, or molecular markers that can reliably distinguish between the two functional forms of autophagy in response to anti-cancer therapies.^66^

The main factors determining which form of autophagy is dominant include two aspects. One is the “molecular switch” that determines the phenotypic transformation of autophagy, such as calcitriol or 1,25-dihydroxyvitamin D3, which is the hormone-active form of vitamin D.^66^ Wilson et al found that when ATG5/7 was lowly expressed, the level of cytoprotective autophagy decreased and the radiation sensitivity of tumor cells increased. After adding 1,25-dihydroxyvitamin D3, the radiosensitizing effect weakened, and the cytoprotective autophagy transformed into cytotoxic autophagy. The other is that the tumor microenvironment has a key regulatory role in the type of autophagy, such as the acidic environment of the stomach.^67^ Zhang et al found that cancer cells overexpressed acid-sensing ion channels (ASIC) and induced autophagy mediated by the autophagy-related gene ATG5, maintaining mitochondrial metabolism and promoting the survival of GC cells.

In addition, the tumor microenvironment can cause GC cells to become resistant to DDP by affecting autophagy. When autophagy is cytotoxic, it may inhibit GC through the following ways: i) eliminating oncogenic proteins such as p62, toxic unfolded proteins, and damaged organelles/DNA; ii) reducing necrosis and local inflammation within the tumor; iii) removing damaged mitochondria (a key source of ROS) to alleviate oxidative stress.^68^^,^^69^ When functioning cytoprotectively, autophagy safeguards GC cells from damage induced by nutrient deprivation, radiotherapy, or chemotherapy. Additionally, it enhances immune evasion, metabolic adaptation, and proliferative capacity in GC cells while promoting angiogenesis, ultimately contributing to drug resistance.^17^

The research by Wang et al emphasized that sodium orthovanadate reduced the level of autophagy by blocking the radiation-induced autophagy-related protein ATG10, thereby increasing the radiosensitivity of GC cells.^70^ Additionally, the study by Lu et al indicated that Withaferin A, a bioactive compound, when combined with radiation, could disrupt the autophagy flux in GC cells, manifested as impaired lysosomal degradation and accumulation of autophagy substrate SQSTM1/p62. This interference might make them more sensitive to radiation-induced damage.^71^

Furthermore, the combination of autophagy and immunotherapy also holds great potential in the treatment of GC. The study by Guo et al demonstrated that using chloroquine to inhibit autophagy promoted the polarization of tumor-associated macrophages to the M1 type, effectively inhibiting tumor cell proliferation, thereby enhancing the chemotherapeutic sensitivity of tumor cells to DDP.^72^ Other studies have shown that autophagy can affect antigen processing and presentation in dendritic cells, and blocking autophagy can further enhance the anti-tumor T cell response, while also having a synergistic effect with immune checkpoint blockade.^11^

In addition to regulating autophagy with radiotherapy and immunotherapy, numerous drugs targeting autophagy have been developed and implemented in clinical practice, including diclofenac, glycyrrhizin, baicalein, omeprazole, chloroquine, metformin, and ubenimex. These agents can sensitize GC cells to DDP by regulating autophagy, thereby enhancing the therapeutic efficacy for GC patients. Furthermore, some potential therapeutic drugs or targets regulate autophagy in DDP-resistant GC cells, enhancing their sensitivity through various mechanisms (Table 2). Examples of such agents include embryonic stem cell-expressed Ras (ERas), RAB12, and CD133. This section will provide a comprehensive examination of how these drugs, along with potential therapeutic agents or targets, affect DDP resistance in GC by modulating autophagy. This underscores the critical need for deeper insights into the role of autophagy and the development of more targeted therapies to address DDP resistance effectively.

**Table 2:** Drugs and molecules that target autophagy to regulate cisplatin resistance in gastric cancer cells.

Agents	Category	Mechanism	Autophagy activator (+) or inhibitor (−)	Sensitivity of cisplatin increased (+) or decreased (−)	Reference
Diclofenac	Drug	Down-regulates the Nrf2 transcription factor in gastric cancer cells and the expression of antioxidant enzymes and AKRC	+	+	79
Glycyrrhizin	Drug	Up-regulates the expression of LC3B and Beclin-1	+	+	82
Baicalein	Drug	AKT/mTOR and Nrf2/Keap 1 pathways	+	+	85
Cucurbitacin B	Drug	Inhibits the CIP2A/PP2A/mTORC1 signaling axis	+	+	90
Metformin	Drug	Activates AMPK phosphorylation	+	+	2
α-mangosteen	Drug	Inhibits the EBI3/STAT3 pathway	+	+	96
Red ginseng polysaccharide	Drug	Inhibits the PI3K/AKT pathway by down-regulating the expression of AQP3	+	+	94
KLF5	Target	Up-regulates the expression of AQP3	+	–	21
CD133	Target	Activates the PI3K/AKT/mTOR pathway	+	–	137
CLIC1	Target	Inhibits the mTOR pathway	+	–	143
MGMT	Target	Increases ATG4B	–	+	139
AKR1C1	Target	ROS accumulation	+	+	109
AKR1C3	Target	ROS accumulation	+	+	109
miR-30b	Target	Targets ATG6	+	+	148
miR-618	Target	Targets Bcl-2	+	+	148
miR-136	Target	Targets Bcl-2	+	+	148
miR-149a	Target	Targets Bcl-2	+	+	148
Bortezomib	Drug	Induces ERK phosphorylation	–	+	125
Chloroquine	Drug	Down-regulates the expression of AQP3	–	+	93
ASII	Drug	Activates the PI3K/AKT/mTOR pathway	–	+	111
All-trans retinoic acid	Drug	Up-regulates the expression of miR-30a	–	–	128
Propofol	Drug	Down-regulates the expression of lncRNA MALAT1, inhibit ATG5 through miR-30e	–	+	109
Ubenimex	Drug	Inhibits the activation of the CD13/PI3K/AKT/NF-κB pathway	–	+	120
Bafilomycin A1	Drug	Up-regulates the expression of LINC-PINT	–	+	44
3-methyladenine	Drug	Inhibits protective autophagy	–	+	114
miR-181a	Target	Down-regulates the expression of ATG5	–	+	130
HOTTIP	Target	Up-regulates the expression of Bcl-2 by miR-216a-5p	–	+	98
circ-PVT1	Target	Targets the Hippo pathway by down-regulating the expression of miR-30a-5p	–	–	99
ERas	Target	Activates the AKT/mTOR pathway	–	+	102
CCL2	Target	Activates the PI3K/AKT/mTOR pathway	–	–	108
METase	Target	Down-regulates the expression of lncRNA HULC	–	+	131
CircMCTP2	Target	Up-regulates the expression of MTMR3 by adsorptive miR-99a-5p	–	+	132
miR-148a-3p	Target	Down-regulates the expression of RAB12	–	+	46

Notes: AKR1C1, aldo-keto reductase1C1; AKR1C3, aldoketoreductase 1C3; ASII, Astragaloside II; CCL2, chemokine C–C chemokine ligand 2; circ-PVT1, circRNA-plasmacytoma variant translocation 1; CLIC1, chloride intracellular channel protein 1; ERas, embryonic stem cell-expressed Ras; KLF5, Kruppel-like factor 5; MGMT, O6-methylguanine-DNA methyltransferase; miR, microRNA; Propofol, 2, 6-diisopropyl phenol.

## Enhancing cytotoxic autophagy to promote GC cell sensitivity to DDP

### Existing drugs that enhance cytotoxic autophagy to promote GC cell sensitivity to DDP

Numerous existing drugs, including diclofenac, glycyrrhizin, baicalein, Cucurbitacin B, red ginseng polysaccharide, and α-mangosteen, have been utilized to enhance the cytotoxic autophagy of GC cells, thereby promoting their sensitization to DDP. Below, we detail these drugs and their respective mechanisms of action.

Diclofenac is a widely used non-steroidal anti-inflammatory drug.^73^^,^^74^ The anti-cancer effects of diclofenac are primarily attributed to its inhibition of the cyclooxygenase enzymes COX-1 and COX-2,^75^ as well as its COX-independent actions that reduce c-MYC expression, subsequently leading to decreased lactate production, glucose uptake, and glutamine breakdown.^76^, ^77^, ^78^ DDP induces excessive ROS through apoptosis and autophagy, ultimately resulting in the death of GC cells. Research conducted by Phoo et al demonstrated that diclofenac down-regulated the nuclear factor erythroid 2-related factor 2 (Nrf2) transcription factor in GC cells, decreased the expression of antioxidant enzymes such as aldo-keto reductases (AKRs), and regenerated intracellular ROS, thereby activating cytotoxic autophagy and enhancing the cytotoxic effect of DDP on KATO/DDP cells, a signet ring cell gastric carcinoma cell line. Moreover, diclofenac mitigated DDP-induced resistance in part by down-regulating cell survival proteins, including Bcl-2, B-cell lymphoma-extra-large (Bcl-xL), and cyclin D1, while also diminishing the activation of mitogen-activated protein kinases (MAPKs), AKT, nuclear factor kappa B (NF-κB), and signal transducers and activators of transcription 3 (STAT3).^79^

Glycyrrhizin is a traditional Chinese medicine derived from licorice.^80^^,^^81^ Wei et al demonstrated that the combination of glycyrrhizin and DDP in the treatment of GC effectively inhibited cell proliferation by blocking the G0/G1 cell cycle. Additionally, this combination enhanced cytotoxic autophagy and promoted apoptosis in DDP-resistant SGC-7901 (SGC-7901/DDP) cells by increasing the cleavage of caspase-8, caspase-9, and caspase-3, as well as the expression of LC3B and Beclin-1, demonstrating significant therapeutic potential against DDP-resistant GC cells. Furthermore, *in vivo* experiments revealed that glycyrrhizin inhibited the transplantation of GC cells in nude mice, confirming its ability to enhance cytotoxic autophagy and improve the sensitivity of GC cells to DDP, thereby supporting its role as an adjunctive therapy with DDP in the treatment of GC.^82^

Baicalein, a bioactive flavonoid, exhibits various pharmacological activities, including antioxidant, anti-inflammatory, and anti-cancer properties.^83^ A prior study by Yu et al suggested that baicalein could overcome DDP resistance in human lung adenocarcinoma cells by inhibiting the PI3K/AKT/NF-κB pathway, and reducing NF-κB-mediated apoptosis in DDP-resistant A549 (A549/DDP) cells.^84^ Recently, Li et al confirmed that baicalein also induced apoptosis and cytotoxic autophagy in GC cells via the AKT/mTOR and Nrf2/Kelch-like ECH-associated protein 1 (Keap1) pathways, thereby enhancing GC cells' sensitivity to DDP.^85^

Cucurbitacin B is a natural compound derived from the Cucurbitaceae family.^86^ It exhibits significant therapeutic effects against cancer, diabetes, neurodegenerative diseases, and various other conditions.^87^^,^^88^ Puustinen et al reported that the cellular inhibitor of protein phosphatase 2 catalytic subunit alpha (PP2A) (CIP2A)/PP2A/mTORC1 signaling axis was pivotal in promoting cell growth while inhibiting cytotoxic autophagy. They discovered that CIP2A bound to mTORC1, functioning as a mutant-resistant inhibitor of mTORC1-associated PP2A, which enhanced mTORC1-dependent growth signaling and suppressed cytotoxic autophagy.^89^ Liu et al demonstrated that cucurbitacin B inhibited the CIP2A/PP2A/mTORC1 signaling pathway, thereby attenuating its suppression of cytotoxic autophagy, and suppressing the proliferation of DDP-resistant GC cells. Consequently, cucurbitacin B effectively enhances the sensitivity of GC cells to DDP and serves as a viable treatment option for GC.^90^

Red ginseng polysaccharide, an active component of Panax ginseng CA Meyer (Araliaceae), is extensively utilized in medicine and exhibits promising activities against cancer cells.^91^ AQP3, a peroxide channel protein, is implicated in the malignant progression of various tumors.^92^ Dong et al found that AQP3 was highly expressed in GC tissues, promoting the conversion of LC3-I to LC3-II in AGS cells, up-regulating the expression of ATG5 and Beclin-1, and down-regulating P62 expression. This process inhibits autophagy and contributes to DDP resistance in GC cells.^93^ Wang et al demonstrated that red ginseng polysaccharide effectively induced ferroptosis in AGS cells in a dose-dependent manner while inhibiting the PI3K/AKT pathway through down-regulation of AQP3. This down-regulation may reduce AQP3-mediated drug resistance, promote cytotoxic autophagy, and enhance the sensitivity of GC cells to DDP. Furthermore, the previously mentioned autophagy inhibitor chloroquine can also mitigate AQP3-induced DDP resistance and improve the sensitivity of GC cells to DDP.^94^

Additionally, α-mangosteen, an antioxidant compound derived from mangosteen peel,^95^ has been shown to enhance the chemosensitivity of SGC-7901/DDP cells to DDP by promoting cytotoxic autophagy and inhibiting the Epstein–Barr virus-induced gene 3 (EBI3)/STAT3 signaling pathway. These findings offer novel insights for combination therapy involving DDP in patients with GC.^96^

Although promoting cytotoxic autophagy can increase the sensitivity of GC cells to DDP, further research is needed to elucidate the underlying mechanisms, determine optimal dosages, and assess the potential side effects of these drugs when used in conjunction with DDP. Clinical trials are essential to validate the efficacy and safety of these therapeutic strategies for patients with GC.

### Potential drugs/targets that enhance cytotoxic autophagy to promote GC cell sensitivity to DDP

In addition to existing pharmacological agents, various other molecules possess the potential to enhance cytotoxic autophagy in GC cells, thereby improving their resistance to DDP. These prospective therapeutic agents and targets encompass a diverse array of substances, including proteins and nucleic acids.

miRNAs are a class of small non-coding RNA molecules ranging from 21 to 25 nucleotides in length. Autophagy mediated by miRNAs, which target numerous molecules, represents a significant mechanism influencing chemotherapy resistance in tumor cells.^97^ Zangouei et al have summarized miRNAs associated with DDP resistance and elucidated that miRNAs are primarily involved in the response of GC cells to DDP by regulating signaling pathways, autophagy, and apoptosis. Among these, miR-30b down-regulates ATG5 to enhance cytotoxic autophagy and inhibit DDP resistance in GC cells. Circ-CCDC66 down-regulates the expression of BCL-2, a negative regulator of apoptosis, by inhibiting miR-618. Additionally, miR-136 and miR-149a also down-regulate the expression of Bcl-2 and AEG1. Thereby, all three miRNAs could reduce the resistance of GC cells to DDP.^97^ Zhao et al demonstrated that the HOXA transcript at the distal tip (HOTTIP) acts as an endogenous “sponge” for miR-216a-5p, which promotes Bcl-2 expression. This process facilitates the formation of the Bcl-2–Beclin-1 complex and increases Beclin-1 content, ultimately leading to a reduction in cytotoxic autophagy in GC cells and decreased sensitivity to DDP. These findings indicate that targeting HOTTIP represents a viable therapeutic strategy to mitigate chemotherapy resistance in GC.^98^ Furthermore, Yao et al found that exosomal circRNA-plasmacytoma variant translocation 1 (circ-PVT1) could target the Hippo pathway by down-regulating miR-30a-5p. This down-regulation decreased the expression of Yes-associated protein 1 (YAP1), a key regulator of downstream target genes in the Hippo pathway. As a result, cytotoxic autophagy in GC cells was inhibited, reducing their sensitivity to DDP. Researchers could explore the use of circ-PVT1 inhibitors to enhance the sensitivity of GC cells to DDP by targeting the circ-PVT1/miR-30a-5p/YAP1 regulatory axis.^99^

ERas is an oncogene that facilitates the tumorigenic growth of embryonic stem cells. Previous studies indicate that ERas can activate both the PI3K/AKT and PI3K/mTOR signaling pathways.^100^ Additionally, the ERas/PI3K pathway enhances cellular resistance to chemotherapy and promotes the proliferation and migration of GC cells, demonstrating characteristics akin to cancer stem cells.^101^ Research by Tian et al revealed that ERas in GC cells inhibited cytotoxic autophagy in the BGC-823 and AGS GC cell lines by activating the AKT/mTOR signaling pathway. Specifically, ERas inhibited cytotoxic autophagy to increase DDP resistance in GC cells, an effect that can be mitigated by autophagy inducers such as rapamycin.^102^

Chemokines represent a family of small cytokine molecules.^103^ Notably, C–C chemokine ligand 2 (CCL2) and its corresponding receptor C–C chemokine receptor 2 (CCR2) have garnered significant attention across various cancers.^104^ Numerous studies have demonstrated that the CCL2–CCR2 axis, a major chemokine signaling pathway, is implicated in the progression of glioblastoma multiforme, as well as various metabolic disorders and depression.^104^, ^105^, ^106^, ^107^ Xu et al discovered that autocrine CCL2 suppressed cytotoxic autophagy, contributing to drug resistance in GC cells, while simultaneously activating the PI3K/AKT/mTOR signaling pathway to up-regulate the autophagy receptor member sequestosome 1 (SQSTM1), thereby enhancing cytoprotective autophagy. The increased SQSTM1 expression subsequently promoted CCL2 expression via the NF-κB signaling pathway, establishing a positive feedback loop that exacerbates drug resistance in GC cells. Consequently, the inhibition of cytotoxic autophagy by CCL2 resulted in chemoresistance, suggesting that autophagy inducers should be employed to counteract the effects of CCL2 and enhance the sensitivity of GC cells to DDP.^108^

Additionally, the enzymes aldo-keto reductase 1C1 and 1C3 (AKR1C1 and AKR1C3) can modulate DDP-induced cell death in KATO/DDP cells by regulating redox-dependent autophagy. The primary mechanism involves the inhibition of AKR1C1 and AKR1C3, which effectively neutralizes the role of aldehyde ketone reductase in scavenging ROS within KATO/DDP cells. This inhibition leads to ROS accumulation, thereby promoting cell death via cytotoxic autophagy.^109^

**Figure 2:**
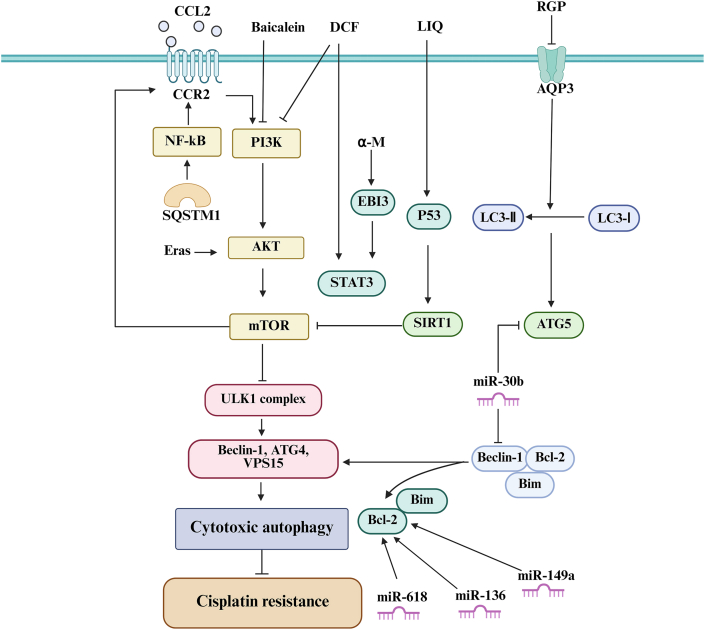
Drugs and molecules that promote cytotoxic autophagy to regulate cisplatin resistance in gastric cancer cells. When autophagy was beneficial to the whole body, CCL2, baicalein, diclofenac (DCF), red ginseng polysaccharide (RGP), and glycyrrhizin (LIQ) could promote cytotoxic autophagy to enhance the killing effect of cisplatin on gastric cancer cells, thereby reducing the resistance of gastric cancer cells to cisplatin. Targeting α-mangosteen (α-M), embryonic stem cell-expressed Ras (ERas), miR-618, miR-136, miR-149a, and miR30b can also play the same role.

In summary, recent foundational and preclinical research has identified various potential drugs and therapeutic targets that may enhance cytotoxic autophagy and mitigate DDP resistance in GC cells. However, further investigations are necessary to translate these findings into clinical applications or to develop novel clinical therapies that target these pathways. We summarize the mechanisms of action of existing and potential therapeutic agents and targets (Fig. 2) to inspire researchers to develop additional drugs that enhance cytotoxic autophagy, thereby improving the sensitivity of GC cells to DDP.

### Inhibiting cytoprotective autophagy to promote GC cell sensitivity to DDP

Given the dual role of autophagy regulation, the resistance of GC cells to DDP can be diminished by inhibiting cytoprotective autophagy. Additionally, a comprehensive overview of current and potential drugs and targets will be presented in the following sections.

### Existing drugs that inhibit cytoprotective autophagy to promote GC cell sensitivity to DDP

Autophagy inhibitors have garnered increasing attention as promising anti-tumor agents. Given that autophagy serves as a protective mechanism, its inhibition may diminish this protective effect in cancer cells, thereby enhancing the drug’s efficacy.^110^ Astragaloside II (ASII), a principal active compound derived from Astragalus, has been shown by Cui et al to significantly inhibit cytoprotective autophagy by affecting lysosomal acidification and enhancing PI3K/Akt/mTOR activity.^111^

Additionally, 3-methyladenine, which acts as both an autophagy inhibitor and a PI3K inhibitor, has been highlighted in previous studies.^112^ For instance, research by Sheng et al indicated that the interaction between 3-methyladenine and chemotherapeutic agents is not necessarily linked to autophagy.^113^ Conversely, Zhang et al utilized both 3-methyladenine and chloroquine to inhibit autophagy, revealing that such inhibition can reverse DDP resistance in SGC-7901/DDP cells.^114^ Consequently, 3-methyladenine emerges as a potential agent to enhance the sensitivity of GC cells to DDP by inhibiting cytoprotective autophagy.

Chloroquine, a 4-aminoquinoline derivative, plays a crucial role in cancer treatment. Chloroquine inhibits the autophagy process by interfering with the fusion of late endosomes and lysosomes.^115^ Dong et al found that chloroquine could partially reverse the chemoresistance to DDP induced by AQP3 overexpression, which may be caused by chloroquine inhibiting AQP3-induced cytoprotective autophagy.^93^ Hou et al reported that chloroquine-mediated autophagy inhibition resulted in the up-regulation of autophagy-related proteins LC3-II/LC3-I and Beclin-1 while decreasing the expression of proteins associated with the mTORC1 pathway. Furthermore, chloroquine decreased the proliferative ability of DDP-resistant GC cells and enhanced apoptosis, thereby increasing the sensitivity of these GC cells to DDP. Consequently, the combination of chloroquine and DDP may represent an effective treatment strategy for DDP-resistant GC.^116^

Omeprazole, a first-generation proton pump inhibitor, reduces gastric acidity by inhibiting gastric acid secretion.^117^ Recent findings suggest that omeprazole induces the silencing of Fat mass and obesity-related protein (FTO) in GC cells and activates the mTORC1 signaling pathway, which inhibits cytoprotective autophagy and enhances DDP-induced apoptosis. Moreover, FTO silencing activated by omeprazole up-regulates the apoptosis-related tumor suppressor gene DNA damage-inducible transcript 3 (DDIT3) through an M6-methyladenosine (m^6^A)-dependent mechanism, promoting tumor cell apoptosis and enhancing the efficacy of chemotherapy drugs against GC cells.^118^

Ubenimex, an inhibitor of the transmembrane glycoprotein CD13 and aminopeptidase N (APN), is widely used as an adjuvant therapy for various cancers.^119^ Guo et al demonstrated that ubenimex inhibited the activation of the CD13/PI3K/AKT/NF-κB pathway, further impeding cytoprotective autophagy, promoting apoptosis, and inhibiting epithelial–mesenchymal transition, ultimately enhancing the sensitivity of GC cells to DDP. Therefore, ubenimex may serve as a promising candidate for increasing the sensitivity of GC cells to DDP.^120^

Bafilomycin A1 is a macrolide antibiotic and a specific inhibitor of vacuolar-type H^+^-ATPase (V-ATPase) in cells.^121^^,^^122^ Bafilomycin A1 is capable of blocking the fusion of autophagosomes and lysosomes, leading to an accumulation of LC3-II that disrupts autophagic flux. Zhang et al applied bafilomycin A1 to drug-resistant GC cells, where they observed that it up-regulated the expression of long intergenic non-protein coding RNA p53-induced transcript (LINC-PINT). LINC-PINT was found to inhibit DDP resistance by reducing cytoprotective autophagy. Furthermore, LINC-PINT recruited the enhancer of EZH2 to the promoter of ATG5, and the overexpression of ATG5 subsequently decreased the level of cytoprotective autophagy. Therefore, they proposed that bafilomycin A1 could inhibit cytoprotective autophagy through the LINC-PINT/EZH2/ATG5 pathway, which ultimately enhances the sensitivity of GC cells to DDP.^44^

Bortezomib is the first-generation proteasome inhibitor approved by the U.S. FDA.^123^^,^^124^ Kao et al demonstrated that the addition of bortezomib blocked cytoprotective autophagy by DDP, and that the combined use of these two drugs synergistically killed cancer cells both *in vitro* and *in vivo*. In drug-resistant ovarian cancer cells, an increase in cytoprotective autophagy was observed. Based on these findings, we hypothesize that the synergistic effect of bortezomib and DDP could yield improved therapeutic outcomes in DDP-resistant GC cells.^125^

In addition, all-trans retinoic acid is a metabolite of vitamin A found in the brain.^126^ All-trans retinoic acid has been shown to exert significant anti-tumor effects in breast cancer.^127^ Abbasi et al demonstrated that all-trans retinoic acid could up-regulate the expression of miR-30a and down-regulate the level of Beclin-1, thereby inhibiting cytoprotective autophagy in GC cells and reducing the cancer stem cell-like characteristics of DDP-surviving cells. This reduction in characteristics enables DDP-resistant G0/G1 phase tumor cells to redistribute to the more sensitive G2/M phase. It is noteworthy that the accumulation of the cell cycle of DDP-surviving cells leads to an increase in DDP-induced cell apoptosis, thereby enhancing the sensitivity of these cells to DDP.^128^ Furthermore, 2,6-diisopropyl phenol (propofol) is a short-acting intravenous anesthetic; numerous studies have indicated that propofol also exhibits effective anticancer properties.^129^ Zhang et al found that propofol down-regulated the expression of lncRNA MALAT1, inhibited ATG5 via miR-30e, and ultimately suppressed cytoprotective autophagy in GC cells while enhancing their chemosensitivity to DDP.^109^

In conclusion, several existing drugs have demonstrated the capacity to inhibit the cytoprotective autophagy of GC cells, thereby increasing their sensitivity to DDP. We propose that numerous other drugs may exhibit similar effects; however, further screening and comprehensive investigation of the underlying mechanisms of action are necessary.

### Potential drugs/targets that inhibit cytoprotective autophagy to promote GC cell sensitivity to DDP

Accumulating evidence indicates that miRNAs play a crucial role in modulating autophagy by regulating the intracellular levels of key autophagy proteins. Zhao et al demonstrated that miR-181a specifically targets ATG5, an essential protein for autophagy, thereby inhibiting cellular autophagic activity. The up-regulation of miR-181a and down-regulation of ATG5 expression significantly enhanced the sensitivity of SGC7901/DDP cells to DDP. These results underscore the potential therapeutic significance of modulating miR-181a and its target ATG5 to enhance the efficacy of DDP in GC.^130^

In addition to miRNAs, other non-coding RNAs, such as lncRNAs and circRNAs, are also recognized as important regulators of autophagy, playing a significant role in counteracting DDP resistance in GC. Xin et al elucidated the mechanisms by which methioninase (METase) regulates autophagy and DDP resistance in GC cells. METase down-regulates the expression of lncRNA HULC and the protein levels of forkhead box M1 (FoxM1), leading to the inhibition of cytoprotective autophagy in drug-resistant GC cells. Additionally, the METase/lncRNA HULC/FoxM1 pathway mediates autophagy-dependent DDP resistance *in vitro*, while the knockdown of HULC results in reduced tumor volume and cytoprotective autophagy *in vivo*. This study highlights the potential of targeting the METase/lncRNA HULC/FoxM1 pathway to overcome DDP resistance in GC.^131^ Previous studies have shown that circMCTP2 expression is down-regulated in DDP-resistant GC cells. Reports indicate that circMCTP2 up-regulates myotubularin-related protein 3 (MTMR3) expression through the adsorption of miR-99a-5p, thereby influencing mTOR activity, decreasing cytoprotective autophagy in DDP-resistant GC cells, and enhancing the sensitivity of GC cells to DDP. Consequently, cytoprotective autophagy inhibition may be one of the mechanisms by which circMCTP2 mitigates DDP resistance in GC cells. The development of drugs aimed at up-regulating circMCTP2 may represent a novel therapeutic strategy to enhance the sensitivity of GC cells to DDP.^132^

RAB12, a member of the RAS oncogene family, influences the levels of autophagy in cancer cells.^133^, ^134^, ^135^ Specifically, RAB12 promotes cytoprotective autophagy and accelerates the maturation of autophagosomes by inhibiting mTORC1 activity, which in turn mitigates DDP-induced cell death. However, Li et al demonstrated that miR-148a-3p significantly decreased autophagic flow and autophagosome formation by modulating RAB12 expression. This finding suggests that miR-148a-3p may serve as a candidate for treating DDP resistance in GC cells.^46^

CD133 is a glycoprotein with five transmembrane domains; its C-terminus and two small loops reside in the cytoplasm, while its N-terminus and two large loops are positioned outside the cell.^136^ Lu et al identified that CD133 activated the PI3K/AKT/mTOR signaling pathway, thereby enhancing the proliferation, anti-apoptotic properties, and cytoprotective autophagy of GC cells. Moreover, it increased the DDP resistance of gastric cancer stem cells. These results indicate that the knockdown of CD133 diminishes cytoprotective autophagy in GC cells. Thus, developing drugs that target CD133 holds promise for treating DDP-resistant GC patients in the future.^137^

O6-methylguanine-DNA methyltransferase (MGMT), an essential DNA repair enzyme, is known to significantly contribute to chemotherapy resistance.^138^ DDP may increase ATG4B by inhibiting MGMT, thereby inducing cytoprotective autophagy in GC cells. This finding was further corroborated by *in vitro* experiments. Future therapies targeting the MGMT–ATG4B–autophagy signaling pathway should incorporate autophagy inhibitors to augment the sensitivity of GC cells to DDP.^139^

Chloride intracellular channel protein 1 (CLIC1), a transmembrane protein that belongs to the CLIC family, is widely expressed in mammalian tissues and cells.^140^ It is closely associated with the high proliferation, active migration, and invasion of tumor cells into non-tumor tissues, thereby establishing CLIC1 as a promising therapeutic target for various cancers.^141^^,^^142^ Nong et al demonstrated that the overexpression of CLIC1 in GC cells induced DDP resistance by activating cytoprotective autophagy through the mTOR pathway. Consequently, the application of CLIC1 inhibitors may enhance the sensitivity of GC cells to DDP, providing novel avenues for the treatment of GC.^143^

Kruppel-like factor 5 (KLF5) is a member of the KLF family and plays a critical role in regulating the expression of numerous genes associated with various cellular functions.^144^ These functions significantly contribute to cancer progression.^145^ Research indicates that poorly differentiated GC subtypes exhibit elevated levels of KLF5 expression, correlating with poor patient prognosis.^146^ Dai et al demonstrated that the zinc finger transcription factor KLF5 up-regulated AQP3 expression, activated cytoprotective autophagy in GC cells, and enhanced resistance to DDP. Consequently, the development of KLF5 inhibitors may suppress GC-related cytoprotective autophagy and further improve the sensitivity of GC to DDP, thereby offering novel avenues for drug development in GC.^21^

**Figure 3:**
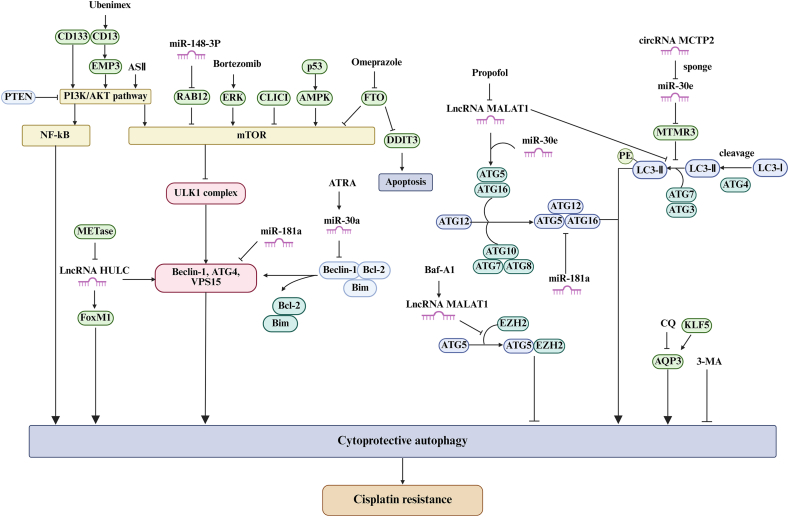
Drugs and molecules that inhibit cytoprotective autophagy to regulate cisplatin resistance in gastric cancer cells. When autophagy acts as a protective mechanism in gastric cancer cells, ASII, 3-methyladenine (3-MA), chloroquine, omeprazole, ubenimex, bafilomycin A1 (Baf-A1), bortezomib, and all-trans retinoic acid (ATRA) can inhibit the cytoprotective autophagy and enhance the killing effect of cisplatin on gastric cancer, thereby reducing the resistance of gastric cancer cells to cisplatin. Targeting miR-181a, lncRNA HULC, circMCTP2, RAB12, CD133, MGMT, CLIC1, and KLF5 can also play the same role.

In conclusion, these potential drugs and targets not only provide hope for new clinical treatments but also highlight mechanisms of action of existing and prospective therapeutic agents, to inspire researchers to develop additional autophagy-inhibiting drugs to enhance the sensitivity of GC cells to DDP. Lastly, we comprehensively summarize the drugs and targets that inhibit cytoprotective autophagy to regulate DDP resistance in GC cells in Figure 3.

Notably, metformin is a unique pharmaceutical agent that exhibits a dual role in modulating autophagy, thereby influencing the sensitivity of GC cells to DDP. The dosage of metformin significantly impacts its mechanism of action. Specifically, metformin can enhance cytotoxic autophagy, which promotes the sensitivity of GC cells to DDP. At concentrations of 25 and 50 mM,^147^ metformin activates p-AMPK in DDP-resistant GC cells, ultimately inducing autophagic cell death and thereby increasing the sensitivity of these cells to DDP.^2^ Fang et al demonstrated that metformin elevated the levels of autophagy markers such as ATG-5, ATG12, and LC3-II by up-regulating acidic vesicular organelles (AVO) and apoptosis-related signaling molecules.^147^ This finding substantiates the notion that metformin enhances the autophagic activity of GC cells through the modulation of lysosomal function, resulting in heightened sensitivity to DDP. Conversely, metformin can also enhance cytoprotective autophagy in a manner that inhibits the sensitivity of GC cells to DDP. Xiao et al reported that metformin activated AMPK signaling through the phosphorylation of AMPK at Thr172 during GC treatment, which subsequently increased mitochondrial autophagy in GC cells and promoted drug resistance.^2^ Finally, we summarize drugs and molecules that target autophagy to regulate DDP resistance in GC cells in Table 2.

## Conclusions and future perspectives

The regulation of autophagy in alleviating resistance to DDP in GC has garnered substantial attention in recent years. Studies have clarified the dual role of autophagy in GC, revealing that inhibiting autophagy can increase the sensitivity of cancer cells to DDP, while in certain circumstances, this relationship may be reversed. Numerous proteins, genes, and signaling pathways linked to autophagy have been implicated in this dual effect. A variety of existing drugs have been utilized to target these proteins, genes, and pathways, thereby modulating autophagy to enhance the sensitivity of GC cells to DDP. Moreover, novel drugs that leverage relevant autophagy regulatory mechanisms may also be developed. As a result, the regulation of autophagy offers a promising strategy for boosting DDP sensitization in GC cells.

Future research is anticipated to focus on optimizing combination therapies that integrate autophagy modulation with DDP. The investigation of additional agents that may synergistically enhance the efficacy of autophagy modulators is an active area aimed at overcoming drug resistance. Furthermore, the development of more selective and potent autophagy modulators could provide improved therapeutic options.

Regarding the identification of autophagy-related drugs or targets, in addition to traditional approaches, there are various approaches such as genetic techniques (*e.g.*, mutations), computational methods (*e.g.*, virtual screening), molecular techniques (*e.g.*, CETSA), and mass spectrometry-based proteomics methods (*e.g.*, LiP-MS). However, these conventional techniques often neglect the dynamic interactions between the target and its microenvironment. Traditional methodologies struggle to analyze complex, dynamic biological networks, often causing targets validated *in vitro* to fail *in vivo* due to intricate regulatory mechanisms, hampering clinical translation. To address this, advanced large language models like GPT-4 offer innovative solutions. Yuan et al have demonstrated that optimizing large language models through prompt engineering (simple prompts, templated prompts, ICL, and multi-round interaction) extends their utility beyond gastrointestinal cancer treatment decisions to identifying novel autophagy-related targets in GC. Among these strategies, multi-round interaction proves most reliable and broadly applicable. We propose that multi-round interaction can be applied to autophagy target discovery through the following iterative process: first, the large language models propose a set of candidate targets; second, clinicians provide additional evidence, highlight contradictions, and correct inaccuracies; third, the large language models integrate the feedback and generate a refined target suggestion.^149^

Innovative approaches, such as gene editing techniques like CRISPR-Cas9, may clarify the roles of ATGs in chemotherapy resistance. As researchers progress from preclinical studies to clinical applications, several trials may be initiated to evaluate the safety and effectiveness of autophagy-targeted interventions in conjunction with DDP for GC patients, particularly to identify subgroups that are likely to benefit most. Additionally, as precision medicine evolves, the integration of autophagy regulation with genomic and molecular profiling of GC could lead to more tailored and effective treatment strategies, ultimately enhancing survival rates and quality of life for patients resistant to conventional DDP therapy. Finally, ongoing investigations into the molecular mechanisms of autophagy and its interactions with other pathways involved in cancer cell survival, apoptosis, and drug resistance will deepen our understanding of how best to manipulate this process for therapeutic advantage.

In summary, while significant advancements have been made in understanding and targeting autophagy to address DDP resistance in GC, further research is necessary to translate these findings into effective clinical applications and therapeutic interventions.

## CRediT authorship contribution statement

**Luling Wei:** Writing – original draft, Software. **Yingfei Zhou:** Writing – original draft, Software. **Jiashuo Li:** Writing – original draft. **Hongzhao Qi:** Writing – review & editing, Supervision, Funding acquisition, Conceptualization. **Shasha Wang:** Writing – review & editing, Supervision, Funding acquisition, Conceptualization.

## Funding

This study was supported by the 10.13039/501100007129Natural Science Foundation of Shandong Province, China (No. ZR2024ME020), 10.13039/100007452Wu Jieping Medical Foundation (No. 320.6750.2024-18.66), and Affiliated Hospital of Qingdao University Clinical Medicine+X (Shandong, China) (No. QDFY+X2023104).

## Conflict of interests

The authors declared no conflict of interests.
